# Resilience and Disaster Trends in the Philippines: Opportunities for National and Local Capacity Building

**DOI:** 10.1371/currents.dis.4a0bc960866e53bd6357ac135d740846

**Published:** 2016-09-14

**Authors:** Tilly Alcayna, Vincenzo Bollettino, Philip Dy, Patrick Vinck

**Affiliations:** Dept of Global Health and Population, Harvard Humanitarian Initiative, Cambridge, Massachussets, USA; Dept of Global Health and Population, Harvard Humanitarian Initiative, Cambridge, Massachussets, USA; School of Government, Ateneo de Manila University, Quezon City, Philippines; Dept of Global Health and Population, Harvard Humanitarian Initiative, Cambridge, Massachussets, USA

## Abstract

Introduction: The Philippines is one of the top countries in the world at risk of climate-related disasters. For populations subsisting at the poverty line in particular, but also the nation as a whole, daily lives and wellbeing are routinely challenged. The Philippines government takes disaster risk seriously and has devoted significant resources to build disaster capacity and reduce population exposure and vulnerability, nationally and locally. This paper explores the policy and institutional mechanisms for disaster risk reduction management and research which have been conducted in the Philippines related to disaster preparedness, management and resilience.

Methods: This study draws on direct observations of and conversations with disaster management professionals, in addition to a review of the extant literature on resilience and disaster preparedness, in the Philippines. This is a descriptive study based on a search of mainly peer-reviewed studies but also articles, reports, and disaster risk reduction and response projects in the Philippines. Search words used in various combinations included: Resilience, Philippines, Disaster Preparedness, Community-based, Disaster Risk Reduction, Capacity-building.

Results: Numerous activities in community based resilience and DRR have been identified across the whole disaster continuum. Yet, important gaps in research and practice remain.

Discussion: The Philippines, is a leading regional actor in disaster risk management. However, a full picture of who is doing what, how, where and when on resilience and disaster preparedness does not exist. Consequently there is no single study that compares the impacts and results that different preparedness measures are having in the Philippines. We recommend further research focussed on mapping the network of actors, understanding community perceptions of disaster risk preparedness and resilience, and investigation into the socio-ecological systems of different communities.

## Introduction

An archipelago of over 7,100 islands, the Philippines is the fourth most at-risk country in the world in terms of climate-related natural disasters, such as typhoons, sea level rise, flooding and extreme temperature.^1^ It is one of the top three countries in the world for population exposure and has the largest proportion of capital investment and stock along coastlines.^2^ Already it is estimated that multi-hazard average annual loss for the Philippines is US$7.893 million, which is equivalent to 69 per cent of social expenditure in the country.^2^ The changing nature of meteorological hazards and emergence of the ‘New Normal’ mean that events such as Super Typhoon Haiyan – and the devastating impact it had - can be expected to occur more frequently, intensifying potential losses.^3^^,^ 4 High levels of poverty (25 per cent of the population are living below the national poverty level) and high inequality^5^ result in large demographics being unable to prepare, cope with and recover from disasters. The Philippines government has devoted significant resources to build disaster capacity and reduce population exposure and vulnerability. A focus on the Philippines with its high risk, challenges of poverty and inequality, can serve as a model on how to build resilience and promote disaster risk reduction (DRR).

This paper explores the policy and institutional mechanisms for disaster risk reduction management and research which have been conducted in the Philippines related to disaster preparedness, management and resilience. Here, the term ‘preparedness’ follows the UNISDR definition of “the knowledge and capacities developed by governments, professional response and recovery organizations, communities and individuals to effectively anticipate, respond to, and recover from, the impacts of likely, imminent or current hazard events or conditions”. The definition of resilience is also taken from UNISDR terminology to mean “the ability of a system, community or society exposed to hazards to resist, absorb, accommodate to and recover from the effects of a hazard in a timely and efficient manner, including through the preservation and restoration of its essential basic structures and functions”. It provides an assessment of extant research on the theory and practice of community-based resilience, highlights the gaps in activities being conducted, and finishes by providing recommendations of key priorities for the future of resilience and DRR work in the Philippines, a leading regional actor in disaster risk management.

## Materials & Methods

***Research Questions***

In addition to a scoping study undertaken in the Philippines in September, 2015^6^, this literature review aims to summarise research around the following questions: What are the advantages of looking at resilience through a community lens? What are the policy and institutional mechanisms for disaster risk reduction management in the Philippines? What work has been conducted in the Philippines related to resilience and DRR? Where are the gaps and what is the future of community resilience in the Philippines?

***Secondary data review***

This is a descriptive study based on a search of mainly peer-reviewed studies but also articles, reports, and disaster risk reduction and response projects in the Philippines. Data was collected on disaster-related projects to-date. The review was done using search engines such as Google Scholar and Harvard Library HOLLIS+. Search words used in various combinations included: Resilience, Philippines, Disaster Preparedness, Community-based, Disaster Risk Reduction, Capacity-building.

***Limitations***

Project specific reports by NGOs, mostly found in the grey literature, have limited inclusion as it was beyond the scope of this paper to assess all previous and on-going projects. Rather, this paper seeks to explore current research in resilience and disaster risk management in the Philippines to understand how research is informing disaster risk management policy and practice in the Philippines.

## Findings

***What are the advantages of looking at resilience through a community lens?***

Like resilience, ‘community’ is a popular term that is still loosely defined in the literature.^7^^,^ 8 A group of people living in the same place or sharing similar characteristics may contain numerous internal conflicts and divisions and may not act as a cohesive entity during a disaster, despite the connotations the term ‘community’ conjures.^9^ Nevertheless, measuring resilience at the community level is advantageous. Communities have a unique understanding of the factors that contribute to their ability to resist, absorb and recover from disturbances as well as a direct understanding of the risks that they face. The social norms, social capital and social networks in which individuals are embedded will determine disaster behaviour and the outcomes of a disaster.^10^ Preparedness plans developed internally by communities have been shown to be better than those developed externally by consultants.^11^ In the event of a disaster, neighbours and local peers are inevitably the first responders. Communities are therefore the most effective locus of disaster preparedness activities.

***What are the policy and institutional mechanisms for disaster risk reduction management in the Philippines?***

The Philippines has a strong set of policies, frameworks and plans for disaster risk reduction (DRR), through which work on resilience can be grounded. The key law is the Philippine Disaster Risk Reduction and Management Act of 2010 (DRRM Law). The DRRM Act establishes local councils at the regional, provincial, municipal, and community levels that replicate the National Disaster Risk Reduction and Management Council's (NDDRMC) responsibilities; however, these local councils are often understaffed or lacking professionalisation and a significant gap exists as the NDRRMC cannot supervise all the local councils.^12^ Local political leaders' support of disaster management, local appreciation of the importance of disaster management, funding, and training and support from the national government determine the effectiveness of local councils.^12^ Climate change is altering the playing field as areas that had historically not been affected by disasters, and as such had been less likely to proactively view disaster management, are increasingly likely to face extreme, unpredictable weather events.^12^

***What work has been conducted in the Philippines related to resilience and DRR?***

*Hazards, vulnerability and risk assessments*

It is uncertain how well disaster risk is communicated to the public, how many projects focus on improving community knowledge on hazards and disaster risk, and challenges remain in measuring and assessing the complex nature of all the factors which can influence disaster risk locally. There are limited studies to measure the combined socio-ecological resilience of the Philippines, at local and national scales,^13^^,^ 14 to help decision-makers locate areas of high vulnerability. Comprehensive risk and vulnerability nation-wide and localised mapping exists from organisations such as the Manila Observatory and the Department of Science and Technology. Post-disaster assessments exist^15^ but there is more need for equivalent *pre-disaster *risk assessments to be generated and shared with communities. Communicating risk information and ensuring communities personalise their risk are proving challenging. Even amongst highly educated demographics, such as medical students, there was a tendency to overestimate the risk of low probability, high consequence disasters such as geophysical disasters (e.g. earthquakes) over high probability events like floods.^16^ Post-Haiyan surveys found that the public had not understood what “storm surge” signified,^17^ did not necessarily know that their houses were located in a potential storm surge area, and even expressed opinions that the risk maps may be exaggerated.^18^ A number of NGOs, including the Philippines Red Cross, conduct community-based vulnerability assessments to improve community awareness. More work on hazard sensitisation and continuing to augment awareness and knowledge of hazards and the threats they pose appear to be needed.

*Early warning systems and evacuations*

Early warning systems and evacuation plans necessarily rely on a public who understands their risks and understand the consequence of the information being disseminated, so that they can prepare appropriately in sufficient time.^18^^,^ 19 ^,^ 20 Community culture, perceptions and values are known to be important components of successful early warning systems and there are calls for greater integration of local/indigenous knowledge related to DRR within science and policy.^21^^,^ 22 Both an independent study and a PAGASA (the Philippine Atmospheric, Geophysical and Astronomical Services Administration) programme introduced community-based monitoring and early warning of hazards into several provinces and showed these were effective complements to traditional centralised early warning systems because they were real-time, localised, empowered those in the best position to undertake preparation and were more likely to be sustained.^23^^,^ 24 Finally, evacuation planning, involving the pre-emptive evacuation of people in high risk locations, has been an effective means of reducing disaster impacts in the Philippines because in general communities are compliant.^15^

*Risk Transfer Mechanisms*

It appears that community networks and reciprocity are the predominant mechanisms through which Filipinos cope with risk. Strong community or familial links have been shown to be just as effective as formal insurance schemes, post-disaster.^25^ On an everyday basis, Filipinos promote *bayanihan*, which is a strong social norm of community welfare and reciprocal labour and comes into play during disasters, in which those less affected help those which have been hit harder.^26^ There is some indication that in geographical regions most exposed to disaster risk, mutual associations and networks devoted to mutual assistance proliferate most readily.^26^ However, community-based mutual assistance activities cannot always be relied upon. Community support may be widespread during the initial rehabilitation efforts, but during long-term recovery community-level activities become rarer and support is exclusive to extended family members.^27^ Community-based activities are nuanced, social networks will be influential and the nature of the disaster and devastation will likely determine how the community comes together and who is excluded.

*Capacity building for disaster preparedness*

Capacity building is occurring across levels from local to national in the Philippines, but focus is predominantly at the local level where numerous actors and networks are collaborating with communities to identify existing capacities, as well as provide the opportunity to build infrastructure, which could minimise the impacts of a hazard.^28^ Differences in community resources, livelihoods options and assets affect local capacity and the extent to which capacity can be strengthened.^29^ A case study in Iloilo City showed that community-driven DRR required strong social networks, alternative finance facilities, technical professional networks that support community processes, and community managed information systems.^30^ Furthermore, it has been highlighted that schools and student groups could play an important, though yet untapped, role in capacity-building for DRR.^31^

The government is also contributing significantly to capacitating local government units (LGUs) by developing a checklist of actions to be taken, supplies to be procured, and important resources together with providing communications and contingency templates for disaster preparedness. These are aimed at the Mayors^32^ as well as local chiefs of police and fire marshals. Yet, it is not clear whether these data collection efforts at the LGU level will contribute to improved national disaster preparedness. Further, there are limits to some of these capacity building projects including:

- "LGUs usually do not demand or procure research and analysis to inform their policy decision-making process on DRR"^33^
- LGU municipalities and barangays lack up to date and sufficient contingency plans^34^
- Political leaders lack adequate DRR training^12^
- Schools have insufficient contingency plans on camp management and preparedness to act as the evacuation centres.^34^

This assessment highlights the continued challenges of transforming policy beyond plans on paper.

*Response and relief operations*

The economic and geographic scale of destruction and damage to infrastructure, housing, communication lines,^34^ and livelihoods assets^35^ tests and often surpasses the national disaster response mechanisms, which otherwise are considered, overall, to “function well”.^12^ Focusing on Typhoon Haiyan, the literature is divided on whether the response was well coordinated or not. On the one hand, the government played an integral role during the response efforts with the international UN cluster system joining the government cluster system and that coordination was good for the most part, resulting in far less morbidity and mortality than previous post-disaster scenarios.^36^ Whilst on the other hand, reports highlight significant tension between the government and INGOs as the L3 response led to the sudden influx of international actors which undermined the usual procedures and relationships established by the Government of the Philippines.^37^

There are cases of different actors working in parallel and duplicating efforts alongside cases of exemplary programming and collaboration. Successful programming included: collaborations between the government and communities for beneficiary selection; organisation of debris from coconut plantations to provide lumber for housing reconstruction; and the restoration of communication lines through emergency radio stations and private networks.^34^ Parallel efforts occurred for a number of reasons:

- National NGOs were unaware of the cluster system and the cluster system did not actively engage with non-cluster actors, leading to a failure to engage with local actors.^47^
- One study found that religious organisations, the private sector and local individuals distrusted the local and national government and so avoided collaboration and coordination.^38^
- Coordination lacked between agencies due to the scale of the disaster. Cash transfers - unconditional and conditional - were used by at least 45 international humanitarians agencies reaching 1.4 million affected people, but were difficult to monitor and coordinate, resulting in many families receiving multiple cash transfers, which distorted the market.

Many lessons have been learnt from the response to Typhoon Haiyan, which will hopefully strengthen the national response mechanisms for equivalent future disasters as policies increasingly focus on preventative and proactive approaches to disaster management.^12^

*Rehabilitation, recovery and reconstruction*

Rehabilitation, recovery and reconstruction programmes in the Philippines are hindered by recurrent disasters, a lack of financial resources, and the politicisation of the process. Linking immediate relief with longer-term recovery and disaster risk reduction remains one of the most persistent challenges of the aid sector globally, largely because of continued under-funding of recovery programmes,^36^^,^ 39 confirmed by the post-Typhoon Haiyan experience where less than half of the $788m needed for recovery had been received six months after the disaster.^40^ Long-term post-disaster assessments reveal the numerous gaps and challenges of the recovery process. Health, especially mental health, was overlooked^41^; thousands remained without permanent settlement^38^; millions were once again living in “unsafe” zones^38^; and politicisation of the process affected vulnerable groups such as internally displaced people.^42^ Despite these problems, reported optimism for recovery is high. Optimism is a powerful aspect of coping capacity and the onus is therefore on the government, local and international organisations to stay committed to their promises and to ensure that disaster affected populations do not lose hope and drive to overcome the impacts of disasters.

## Discussion

***Where are the gaps and what is the future of community resilience in the Philippines?***

Numerous activities in community-based resilience and DRR have been identified across the whole disaster continuum. Yet important gaps in research and practice remain. Most noticeably, the extant studies fail to provide a full picture of who is doing what, how, where and when on resilience and disaster preparedness. Lacking this, there is consequently no single study that compares the impacts and results that different preparedness measures are having in the Philippines.

In addition, specific gaps were identified in programming focusing on public knowledge about risks; data collection and socio-ecological research; and understanding communities. Firstly, a changing climate and more extreme weather events mean that communities can no longer rely on past experience to help prepare for future disasters. Communities need to be able to access current continually updated information on what changing global environmental systems and the impacts of previous disasters mean for their future disaster risk. Secondly, important data and research which could help inform policy and disaster management decisions are lacking, including: published data on local, disaggregated environmental and ecological changes and how these changes feed into disaster risk; population-based surveys on disaster risk perceptions and preparations; research on how smaller scale disasters may erode resilience; and long-term recovery and relocation initiatives to ensure transformative adaptation towards greater resilience.^43^^,^ 44 ^,^ 45 ^,^ 46 Thirdly, despite communities being the focus of attention of a number of studies, there are differing definitions of community, varied ways of measuring social capital, and limited research on marginalised persons who are excluded from community support (such as *bayanihan)*.

Many important questions remain to be addressed such as what training and support do local political leaders need so that they are more effective in DRR?^12^ Can communities withstand future Haiyan-like events? What are the limits of community-based disaster resilience? Which community members are likely to be excluded from community networks? What pressures can these networks withstand and under what conditions do they breakdown?

***Recommendations for future work***

To further build on the ongoing disaster preparedness and resilience initiatives occurring in the Philippines, three top priorities for future work were identified:

1. Map the network and activities of national and international agencies and actors working on resilience and disaster preparedness. This mapping should capture who is doing what activities and where. It would help identify programmatic and geographic gaps and overlaps and contribute towards increasing coordination and mutual learning among the different actors.
2. Research into community perceptions of disaster risk preparedness and resilience. Risk perception, cognitive barriers and cultural values shape how people will respond to disaster warnings and preparedness initiatives. Interventions and knowledge campaigns must be tailored to ensure maximum acceptance and adoption by people and their communities. This research is vital to help inform policy, initiatives, and operational programming.
3. Increased research into the socio-ecological systems and what metrics can capture this system. This research must look at how climate change will impact environmental systems which in turn affect social systems; how certain demographics (e.g. informal settlers) may live in different socio-ecological systems compared to their wider communities. This research would help inform mitigation and prevention strategies alongside preparedness.

## Conclusion

This paper assessed the extant research and practice of resilience and disaster preparedness in the Philippines, which serves as a good model on how to strengthen resilience and promote disaster risk reduction at the local level. Research and interventions are already identifying examples of best practice in disaster preparedness, response and recovery; however, important underlying drivers of disaster risk, such as a degrading environment and inequality, still remain over looked. With the frequency and intensity of disasters set to increase, communities are going to have to prepare more for worse events. This poses the question of how much longer we can react to disasters rather than mitigating them in the first place. The urgency with which we must address the research gaps across the disaster cycle, and in particular in preventing and mitigating disaster risk alongside preparedness, is mounting. Research findings must then be translated in policy decisions with committed implementation. A greater prioritisation of mitigation, prevention and preparedness is not only economically advantageous, but from a humanitarian point of view, reduces the human costs, and aligns with initiatives on sustainable development.

## Data Availability

There is no raw data associated with this paper.

## Correspondence

Tilly Alcayna: t.alcayna@gmail.com

Vincenzo Bollettino: vbollett@hsph.harvard.edu
